# A Few Facts about Dental Caries

**Published:** 1905-01-15

**Authors:** Aug. Lohmann, E. C. Kirk


### A FEW FACTS ABOUT DENTAL CARIES.

BY DR. AUG. LOHMANN AND DR. E. C, KIRK.

The author believes that mucin has a decalcifying action upon the enamel of the teeth. He has observed that people whose salivary secretion is abundant, with mucin in large proportion, have a considerable number of carious teeth, and that the carious process is more rapid when the saliva is decidedly viscid. He has carried on a series of investigations and found that mucin has as strong a decalcifying action as lactic acid. A molar weighing 2.131 gm. suspended in the mucin obtained from the saliva of a girl aged eighteen weighed only 2.112 gm. at the end of the thirtieth day. The saliva of pregnant women contains a great proportion of mucin and is therefore more decalcifying than that of the normal subject. A tooth weighing 2.066 gm. suspended in the saliva of a pregnant woman weighed only 1.777 gm. after thiry days' sojourn in the fluid. An experiment was made with lactic acid in 2 percent solution. A tooth weighing 2.117 gm. was allowed to remain in this solution for thirty days. At the conclusion of the experiment it weighed 2.072 gm. It should be remembered that lactic acid is present in the salivary fluid to the extent of 0.75 percent.

The effects of the presence of mucin upon the surfaces of teeth and mucous membrane can be neutralized by the action of slightly astringent and antiseptic solutions, or by means of a physiological salt solution, which is a solvent for mucin.—*Lohman.*

The paper of which the above is a resume, is published in full in *Archiv fur Zahnheilkunde* for June, 1904. The claim is novel, not to say startling, in view of the fact that we have come to be pretty well assured that the problem of the etiology of dental caries has been practically settled through the researches of Miller and those who have confirmed his observations by further research. Indeed, so revolutionary does the claim of Lohmann at first reading appear that one's feeling regarding it is well expressed in the words of Prof. Miller himself in his paper which we publish in this issue, as follows: "The view recently promulgated by Lohmann that caries of the teeth *is due solely to the action* of the mucin of the saliva stands at variance with some of the simplest facts of dental pathology and bacteriology to such an extent that it hardly seems indicated to enter into a discussion of it here."

In view of the sweeping character of Lohmann's claim the criticism of Miller above quoted does not seem to us to be unwarranted, yet a careful reading of Lohmann's paper leads us to feel that in spite of his sweeping generalization he has raised certain questions regarding the relation of mucin to the causation of dental caries which cannot be so summarily dismissed, and which are entitled to thoughtful consideration. We think it has been demonstrated to a finality that dental caries is conditioned upon bacterial activity, and that the dissolution of tooth-structure is in the first place brought about by acids, which agents alone have the power to dissolve the calcium phosphate of tooth-structure. It has also been demonstrated that the bacteria concerned in the production of dental caries generate lactic acid as a result of their fermentative action upon carbohydrates, and that the lactic acid so produced dissolves the calcium phosphate of the tooth. It has not, however, been shown that mucin may not be a factor in the process. Lohmann's claim that mucin is capable of directly producing caries, if such be his position, cannot be taken seriously in view of what is already known of the carious process.

The data regarding the properties and composition of mucin to which Lohmann directs attention are, however, of such a character as to be worthy of investigation. The fact that mucin is in itself acid in character would indicate that it has a solvent action *per se* upon tooth-structure. This fact seems to have been verified by Lohmann's experiment, in which he shows comparatively the solvent action of mucin and lactic acid respectively upon teeth. But granted that mucin will dissolve tooth-structure, it must be borne in mind that simple tooth-solution is not tooth-caries; nor can anything even simulating caries be produced in a tooth by the direct application of acid solvents. Chemical erosion might be so produced, but caries is not erosion, and has nothing in common with it so far as its microscopical appearances are concerned. Two facts in connection with Lohmann's contention seem to us to be of importance. First, that caries is more prevalent in mouths characterized by a viscid, stringy saliva; this we think will fully accord with the results of clinical observation, and the viscidity of the saliva we believe is rightly attributable to its abnormal content of mucin. Second, the fact that there is a 25 percent carbohydrate moiety in the chemical composition of mucin, and it would seem not improbable that this carbohydrate feature of mucin might possibly form a fermentable pabulum for the action of the lactic acid group of bacteria through the activities of which tooth-caries might result.

In addition to the points adduced by Lohmann in favor of his mucin theory, it is noteworthy that the saliva of arthritic individuals, who are generally immune from dental caries, is also lacking in mucin—indeed, in many it is almost totally absent as a constituent of the saliva. The question raised by Lohmann is an interesting one. Some of his deductions do not seem to be warranted by the evidence presented and especially in view of what is already known of the causation of dental caries, but there is much about this important question that is still unsolved, especially regarding the fact of the immunity from this disease which is enjoyed by many individuals. And we are more and more impressed with the belief that the chemical composition of the saliva is the key to the situation. Hygiene of the mouth is not sufficient to explain immunity, nor does an unclean mouth explain susceptibility to caries.

Clinical observations refute both possibilities. Something in or something lacking in the composition of the saliva which determines its suitability as a pabulum for the development of caries-producing fungi seems to us to be a guess much nearer to the truth, especially as the variability in composition of the saliva in different diathetic states is in fairly close relation to immunity and susceptibility to caries in various individuals. We know little of the meaning and function of mucin as a constituent of the saliva. It offers an interesting field for study. We should know all that it is possible to know about the composition of the oral fluids, and as a matter of fact we must possess this knowledge before we can settle the question not only of caries but of many other oral conditions. Therefore we feel indebted to Dr. Lohmann for directing attention to this matter, which, if not as important in its bearings as he is inclined to regard it, is at least of sufficient importance to be worthy of careful and systematic study.—Editorial in December *Cosmos*.

